# Interleukin-26 is a promising biomarker of sepsis but is it always reliable?

**DOI:** 10.1186/s13054-019-2691-3

**Published:** 2019-12-06

**Authors:** Patrick M. Honore, Aude Mugisha, Leonel Barreto Gutierrez, Sebastien Redant, Keitiane Kaefer, Andrea Gallerani, David De Bels

**Affiliations:** ICU Department, Centre Hospitalier Universitaire Brugmann, Place Van Gehuchtenplein, 4, 1020 Brussels, Belgium

We read with interest the recent article by Tu et al. concluding that interleukin-26 (IL-26) is a better predictor of 28-day mortality in septic patients when compared with C-reactive protein (CRP) and procalcitonin (PCT) [1]. However, SOFA score remains the best predictor by far over IL-26. While we applaud the results of this study, we would like to make some comments. IL-26 is the most recently identified member of the IL-20 cytokine subfamily and is a promising mediator of inflammation overexpressed in activated or transformed T cells [2]. IL-26 has a molecular weight ranging between 19 and 36 kDa. Nearly half of critically ill patients especially those with septic shock have or develop acute kidney injury (AKI), and 20–25% will need renal replacement therapy (RRT) within the first week of their stay [3]. Out of the 52 septic patients in this study, several patients will develop AKI and necessitate continuous RRT (CRRT) [1]. The serum for IL-26 was taken on admission in the intensive care unit (ICU) in this study, and nothing can be said about the reliability of the admission level. Nevertheless, we would like to warn the clinician about daily monitoring of IL-26 like for CRP and PCT. CRRT is performed using membranes that have a cutoff value of 35–40 kDa, and therefore, some quantity of IL-26 will be eliminated by CRRT [4]. New highly adsorptive membranes (HAM) that can adsorb many molecules with a molecular weight above 35 kDa will even increase this removal [5]. This can mislead patient prognostication by artificially decreasing IL-26, but no studies have challenged this issue yet. Such studies should be done as there is already a long list of biomarkers in sepsis that are lacking reliability during CRRT [5]. To date, no single sepsis biomarker can be reliable during CRRT. While admission levels of IL-26 might be a good marker of severity and mortality of sepsis, this might not be the case for septic shock under CRRT [4, 5].

## Data Availability

Not applicable.

## Abbreviations

IL-26: Interleukin-26
CRP: C-reactive protein
PCT: Procalcitonin
AKI: Acute kidney injury
RRT: Renal replacement therapy
CRRT: Continuous renal replacement therapy
HAM: Highly adsorptive membranes
